# Deciding to End One’s Life Because of a Psychiatric Illness—A Decision Without Second Thoughts?

**DOI:** 10.3389/fpsyt.2020.00058

**Published:** 2020-02-18

**Authors:** Andres R. Schneeberger, Paul Hoff, Erich Seifritz, Undine E. Lang, Marc Graf, Irina Franke, Christian G. Huber

**Affiliations:** ^1^ Klinik für Psychiatrie, Psychotherapie und Psychosomatik, Psychiatrische Klinik der Universität Zürich, Zurich, Switzerland; ^2^ Erwachsenenpsychiatrie, Psychiatrische Dienste Graubünden, Chur, Switzerland; ^3^ Department of Psychiatry and Behavioral Sciences, Albert Einstein College of Medicine, Bronx, NY, United States; ^4^ Universitäre Psychiatrische Kliniken Basel (UPK), Klinik für Erwachsene, Universität Basel, Basel, Switzerland; ^5^ Universitäre Psychiatrische Kliniken Basel (UPK), Klinik für Forensik, Universität Basel, Basel, Switzerland

**Keywords:** physician-assisted dying, bioethics, suicide prevention, psychiatry, suffering

In the September issue of the American Journal of Bioethics, Kious and Battin (1) present their arguments on why physician aid-in-dying (PAD) due to severe suffering should also be allowed in non-terminal psychiatric diseases. The authors argue that a crucial aspect of PAD is the assessment of the decision-making capacity. Furthermore, they elaborate on the incompatibility of current PAD regulations and compulsory treatment because of suicidality, emphasizing differences between European, Canadian, and US-American policies. They differentiate between possible pathways the discussion about laws and policies concerning medically assisted dying could lead to. Firstly, keep the status quo, requiring a terminal illness, without considering the suffering caused by mental illness. Secondly, a change toward a partial opening of PAD for people with mental illness if their decision-making capacity is intact. This approach would require a change in policies regarding assisted dying while at the same time changing the involuntary civil commitment practices. The third approach devises a metric to measure suffering. While allowing patients who reach the threshold of unbearable suffering to access PAD, people with lower scores of suffering would fall under the policies of involuntary civil commitment and treatment. This third approach poses difficult questions concerning the nature of an instrument to determine suffering, the definition of suffering and its thresholds, and about the authority of the gatekeeper determining whether a person qualifies for PAD.

We congratulate the authors for their balanced and differentiated argumentation on a highly important and still critically discussed topic in psychiatry. The line of thought they present focuses on the situation in the United States of America and resembles the current discussion in Switzerland. The history of PAD in Switzerland dates back to the early 1980s and has since then been part of the public and psychiatric discourse (2). The criteria include a terminal illness after all treatment options have failed, a fact which has to be confirmed by a third party. In 2018, the Swiss Academy of Medical Sciences (SAMW) updated their recommendation, replacing the requirement of terminality by the criteria of unbearable suffering. This change of policy would, among others, allow people suffering from mental illness to access PAD (3). The Swiss Medical Association (FMH), however, decided not to adopt this recommendation—a first in the history of the FMH—and to restrict PAD to cases of terminal illness (4). This dispute reflects the change from the first to the second approach discussed by Kious and Battin.

We share the opinion that persons suffering from a psychiatric illness should not automatically be excluded from PAD despite having the capacity to decide in this issue. This can be seen as one of the many instances of structural stigmatization this person group has to endure (5), and it is incompatible with the current motion of empowerment in psychiatry (6). However, the unfinished discourse about the definition and importance of being terminally ill—as it is currently discussed in Switzerland—seems to be a highly relevant aspect that is insufficiently discussed by Kious and Battin. They correctly refute the ideas that terminality is required to minimize the amount of life lost, and that causing the death of someone who is terminally ill is not really killing. Correctly, they conclude that terminality is required as a safeguard. Then—prematurely and without the necessary critical discussion, in our view—they dismiss this point as “question-begging” when balancing the prevention of a slippery slope and the exclusion of a whole group of severely suffering individuals from PAD. The difficulty of this decision is also based on the partially opposing and overlapping theories of suicidality, unbearable suffering, terminality, and irreversibility. The authors do not discuss PAD within the concept of suicidality. It seems crucial to distinguish the symptom suicidality as part of a psychiatric syndrome or disorder in contrast to the wish to die based on unbearable suffering caused by mental illness. To date, terminality has served as a potential safeguard: 1) to ensure the irreversibility and 2) to warrant stability regarding the decision for PAD. The first point assumes that irreversibility can be used synonymously with terminality. However, in the case of mental illness, irreversibility of unbearable suffering might be defined by the lack of therapeutic interventions potentially relieving the symptoms, while not qualifying as a terminal illness. The second argument highlights the contrast, as we know that a considerable percentage of persons who choose to commit suicide regret this decision later on (7). It is even quite common that persons having decided to commit suicide abandon this plan during the act of suicide. This does not necessarily mean that the person has not really made up their mind before acting, that he or she did not really want to die, or that the suicidal act is discontinued because of pain that would not be the case in PAD. It can also be an expression of genuine “second thoughts,” of a change of perspective when directly confronted with the consequence of death. Complicating this issue even more, coming to terms with an irreversibly poor health condition despite unchanging situation and suffering is one of the therapeutic responsibilities in mental health care. Current psychiatry aims to reach recovery even in chronically ill patients (8), and pursuing recovery in chronic suffering due to psychiatric disease may mean that a person can change from embracing PAD to embracing living with their suffering.

Hence, we propose that before starting a dialogue on potential policy and legal changes regarding PAD and involuntary commitment, the discussion should focus on the conditions under which a severe mental illness might lead to such a pronounced and unbearable state of suffering, with no prospect of therapeutic improvement. Rather than finding measures of suffering and defining thresholds, it is important to look at the complexity of human existence while suffering from a mental disorder. Life constellations of people searching the path of PAD need to be assessed, taking into consideration irreversibility in the context of biological, psychological, social, and spiritual characteristics. It is crucial to empower patients, peer workers, and relatives of people with mental illness to participate in this discourse. Only a participative approach will help us find solutions that bridge the gap between the needs of people with mental illness and society as a whole.

## Author Contributions

AS and CH performed the literature search and wrote the first draft of the comment, all other authors (PH, ES, UL, MG, IF) contributed important scientific information to the paper, read and approved the final paper for submission.

## Conflict of Interest

The authors declare that the research was conducted in the absence of any commercial or financial relationships that could be construed as a potential conflict of interest.
